# Real‐world outcomes of aflibercept 8 mg in patients previously treated for neovascular age‐related macular degeneration

**DOI:** 10.1111/aos.17590

**Published:** 2025-09-08

**Authors:** Imadeddin Abu Ishkheidem, Esra Inci, Martin Breimer, Sofia Töyrä Silfverswärd, Madeleine Zetterberg, Marita Andersson Grönlund

**Affiliations:** ^1^ Department of Clinical Neuroscience Institute of Neuroscience and Physiology, University of Gothenburg Gothenburg Sweden; ^2^ Department of Ophthalmology Region Västra Götaland, Sahlgrenska University Hospital Mölndal Sweden; ^3^ Department of Ophthalmology, Faculty of Medicine and Health Örebro University Örebro Sweden

**Keywords:** aflibercept, age‐related macular degeneration, ocular inflammation, optical coherence tomography, real‐world data

## Abstract

**Purpose:**

To evaluate visual, anatomical and safety outcomes of aflibercept 8 mg in previously treated patients with neovascular age‐related macular degeneration (nAMD).

**Methods:**

This retrospective study included nAMD patients switched to aflibercept 8 mg from prior anti‐VEGF therapies at Sahlgrenska University Hospital between February 2024 and February 2025. Data on best‐corrected visual acuity (BCVA), central retinal thickness (CRT), pigment epithelial detachment (PED) height, fluid status, treatment intervals, time to fluid recurrence and adverse events were collected.

**Results:**

181 eyes (167 patients; mean age 80.4 ± 8.5 years; 67.7% female) were included, with a median follow‐up of 12.9 weeks (range 4.1–48.1). A total of 415 injections were administered (mean 2.1 ± 1.5 per eye). BCVA remained stable (baseline 0.46 ± 0.31 logMAR; post‐treatment 0.47 ± 0.37 logMAR; *p* = 0.18). CRT decreased significantly (−19.5 ± 47.2 μm; *p* < 0.001), as did PED height (−37.4 ± 68.4 μm; *p* < 0.001). Intraretinal fluid prevalence decreased from 34.3% to 19.3% (*p* < 0.001) and subretinal fluid from 53.0% to 33.7% (*p* < 0.001). The median maximal dry interval achieved was nine weeks, and analysis of interval extension showed a statistically significant mean increase of 1.27 ± 4.24 weeks overall (*p* = 0.0009), particularly in eyes dry at baseline. The median time to fluid recurrence among those with reactivation was ten weeks. Higher baseline CRT predicted greater CRT reduction (−44.1 μm per 100 μm increase; *p* < 0.001) but shorter dry intervals. Safety was favourable, with one case (0.6% per eye; 0.2% per injection) of mild anterior uveitis and no cases of intraocular pressure elevation.

**Conclusions:**

Switching to aflibercept 8 mg led to stable vision, significant anatomical improvements, extended treatment intervals and a favourable short‐term safety profile. Longer follow‐up is warranted.

## 1 INTRODUCTION

Age‐related macular degeneration (AMD) remains a leading cause of visual impairment globally, with the neovascular form (nAMD) responsible for most cases of significant vision loss (Kawasaki et al., 2010; Klaver et al., 2001; Klein et al., 1999; Mitchell et al., 1995; Wong et al., 2008, 2014). This condition is characterized by abnormal macular neovascularization (MNV), leading to fluid accumulation, haemorrhage and irreversible damage to photoreceptors and the retinal pigment epithelium (Ferris et al., 2013). Central to its pathophysiology is the overexpression of vascular endothelial growth factor (VEGF), which promotes neovascular leakage and proliferation. Placental growth factor (PlGF) has also emerged as a contributor to disease progression (Van Bergen et al., 2019). In addition to VEGF, angiopoietin‐2 (Ang‐2) has also been identified as a therapeutic target in neovascular AMD, as demonstrated in pivotal trials of faricimab (Heier et al., 2022).

The introduction of intravitreal anti‐VEGF agents has revolutionized the management of nAMD. Aflibercept, a recombinant fusion protein, binds VEGF‐A, VEGF‐B and PlGF, preventing these growth factors from interacting with their receptors and thereby inhibiting abnormal vessel growth and leakage. It has demonstrated favourable anatomical and functional outcomes since its approval (Heier et al., 2012). In standard practice, aflibercept is administered at a dose of 2 mg, but frequent injections are required, creating a high treatment burden in real‐world settings (Holz et al., 2015).

To address this challenge, a higher‐dose formulation of aflibercept (8 mg) was developed, aiming to allow for longer dosing intervals while maintaining efficacy. The PULSAR phase 3 trial demonstrated that aflibercept 8 mg, administered at 12‐ or 16‐week intervals, achieved non‐inferior visual outcomes and a similar safety profile compared with the 2 mg dose given every 8 weeks (Lanzetta et al., 2024). Based on these findings, aflibercept 8 mg received approval from the U.S. Food and Drug Administration in August 2023 and from the European Commission in January 2024 for the treatment of nAMD (European Commission, 2024; U.S. FDA, 2023).

While randomized controlled trials (RCTs) provide robust evidence within controlled environments, real‐world data are crucial for evaluating therapeutic performance across broader, more diverse populations and in less structured clinical settings. In routine practice, patients often differ from clinical trial cohorts in terms of age, comorbidities and adherence patterns (Lotery et al., 2017). Against this background, we aimed to evaluate the real‐world use of aflibercept 8 mg in previously treated nAMD patients, with a focus on visual and anatomical outcomes, fluid dynamics and safety.

## 2 METHODS

### 2.1 Study design and setting

This was a retrospective cohort study conducted at Sahlgrenska University Hospital Gothenburg, Sweden, evaluating real‐world outcomes of aflibercept 8 mg in patients with exudative nAMD from February 2024 to February 2025. The research protocol adhered to the ethical standards of the Declaration of Helsinki and received approval from the Swedish Ethical Review Authority (Dnr 2025‐00189‐01).

### 2.2 Participants

Eligible eyes were diagnosed with exudative nAMD and treated with aflibercept 8 mg following a switch from prior anti‐VEGF therapy. Previous treatments included aflibercept 2 mg, faricimab 6 mg, brolucizumab 6 mg or bevacizumab 1.25 mg. One patient had received a dexamethasone 0.7 mg intravitreal implant (off‐label use) at the time of switch, after having received several prior anti‐VEGF injections. All patients were treated within the defined study period at the study site.

Exclusion criteria included the following:

- Incomplete data or absence of follow‐up during the study period
- Transfer of care to another clinic during follow‐up
- No evaluable baseline optical coherence tomography (OCT) before switch to aflibercept 8 mg, including:
  ◦ Baseline OCT performed while the patient was still receiving aflibercept 2 mg (e.g. due to waiting time or drug shortage).
  ◦ Switch to aflibercept 8 mg made without a valid OCT assessment at baseline or follow‐up.
- Mixed use of aflibercept 2 and 8 mg within the same treatment series
- Misinterpretation of macular status on OCT, such as dry macula incorrectly assessed as having fluid (e.g. a small pigment epithelial detachment, PED, mistaken for subretinal fluid or outer retinal tubulation mistaken for intraretinal cysts)
- Macular neovascularization treated with co‐administration of tissue plasminogen activator (Actilyse)
- Underwent cataract surgery during the treatment period
- Best‐corrected visual acuity (BCVA) confounded by treated posterior capsular opacification (PCO) during the study period, making interpretation of BCVA unreliable.

### 2.3 Data collection

Baseline was defined as the time immediately prior to the first aflibercept 8 mg injection. Demographic data (age, sex) and clinical characteristics were extracted from electronic medical records (EMR). BCVA was recorded in Snellen format and converted to logarithm of the minimum angle of resolution (logMAR) for statistical analysis.

Baseline imaging was obtained using swept‐source OCT (Topcon Triton, Tokyo, Japan) and included measurements of central retinal thickness (CRT), presence of intraretinal fluid (IRF), subretinal fluid (SRF) and PED. All types of exudative AMD were included in the study, regardless of MNV subtype. Only fibrovascular PEDs located within the central 1500 μm were analysed to exclude drusenoid or pure serous PEDs that are unlikely to respond to anti‐VEGF treatment and could confound anatomical outcomes. The central 1500 μm region was selected because changes in this area directly affect visual acuity and central retinal thickness.

Prior treatment history was documented, including the most recent anti‐VEGF agent administered, the number of prior intravitreal injections, the number of different agents used before the switch and the treatment interval immediately before switching.

At follow‐up, data collected included BCVA, OCT parameters, number of aflibercept 8 mg injections received, the maximal injection interval achieved with a dry macula and the time to recurrence of retinal fluid when applicable.

### 2.4 Outcome measures

The primary outcome was change in BCVA (logMAR) from baseline to the last follow‐up visit during the study period.

Secondary outcomes included:

- Change in CRT
- Change in PED height
- Fluid resolution at final visit (absence of IRF and/or SRF)
- Maximal treatment interval which was achieved with a dry macula
- Interval extension
- Time to recurrence of retinal fluid
- Number of aflibercept 8 mg injections received
- Adverse events, including intraocular inflammation (e.g. iritis, vitritis, retinal vasculitis), elevated intraocular pressure (IOP) and endophthalmitis, were noted. Routine IOP measurements were not performed after treatment, and this applied to all types of anti‐VEGF therapy; instead, IOP was assessed only if the patient reported symptoms following the injection.

Outcome measures were summarized from baseline to each patient's last available follow‐up visit within the defined one‐year observational period. Patients were recruited successively during the year, leading to variable follow‐up durations depending on enrolment timing. Eyes reswitched to another anti‐VEGF, lost to follow‐up or discontinuing treatment were analysed until their last documented appointment during the observation period.

### 2.5 Treatment and follow‐up protocol

Patients were switched to aflibercept 8 mg for three main reasons:

1. Persistent fluid despite prior therapy, indicating an inadequate response to previous anti‐VEGF treatments.
2. Anatomically dry macula but requiring short injection intervals to maintain dryness, where the switch aimed to enable longer treatment intervals.
3. A centre‐wide protocol change implemented during the study period, replacing aflibercept 2 mg with aflibercept 8 mg, was implemented for logistical efficiency and economic considerations.

Treatment was initiated with two to three injections, with intervals individualized based on OCT findings, disease activity and prior treatment intervals, following a treat‐and‐extend regimen.

### 2.6 Analysis of interval extension and subgroup analyses

To evaluate changes in treatment intervals following the switch to aflibercept 8 mg, we compared the interval length immediately before the switch with the maximal treatment interval achieved with a dry macula after the switch for each eye, calculating interval change as the difference between these two intervals. The Wilcoxon signed‐rank test was used for paired comparisons of pre‐ and post‐switch intervals. To address variability in follow‐up times, a subgroup analysis was performed in eyes with at least 16 weeks of follow‐up, comparing interval extension to the full cohort. Eyes were excluded from interval extension analyses if they had disease reactivation requiring shortened intervals after previously stable long intervals, inconsistent follow‐up schedules or assessments where disease activity evaluation may have been inaccurate, to avoid biasing results with non‐representative data.

### 2.7 Statistical analysis

Descriptive statistics were used to summarize baseline characteristics. Continuous variables were summarized using both mean ± standard deviation (SD) and median (minimum–maximum) to provide an overall understanding of central tendency and spread, regardless of distribution. As no between‐group statistical comparisons were performed, formal normality tests were not conducted. Categorical variables were summarized as counts and percentages. For tests of change in binary variables over time, the Sign test was used, and for continuous variables, the Wilcoxon signed‐rank test was applied. Diagnostic plots of residuals from linear mixed‐effects models were examined to confirm that assumptions of linearity and normality were satisfied.

To assess changes in continuous outcomes such as BCVA (logMAR), CRT and PED height, linear mixed‐effects models were applied. These models accounted for within‐subject correlation in cases of bilateral eye inclusion using random intercepts where convergence permitted. Fixed effects included age, sex and the respective baseline value. In the model where the previous treatment was studied, where data were insufficient and convergence issues occurred, random effects were omitted and only fixed effects were included.

For the analysis of maximal treatment interval with a dry macula, Poisson regression for repeated measures was used, modelling the outcome as a rate. To account for variability in follow‐up duration among patients enrolled throughout the one‐year observational period, we included an offset term for each eye's individual follow‐up time in the Poisson regression models of interval‐related outcomes. This adjustment allowed fair comparisons of maximal dry interval estimates despite differences in observation time. Results were expressed as incidence rate ratios (IRRs), which can be interpreted as the relative risk of having a longer (for RR >1.00) or shorter (for RR <1.0) treatment interval (measured in weeks) per unit change in the explanatory variable.

To validate our findings in patients with longer observation periods, we conducted another subgroup analysis restricted to eyes with at least 12 weeks of follow‐up, comparing interval‐ and fluid‐related outcomes to the overall cohort.

Associations between selected variables and fluid status at the end of treatment (intraretinal, subretinal or any fluid) were evaluated using generalized estimating equations (GEE) with binary outcomes. These models were adjusted for baseline fluid status, age and sex.

Eyes from patients with unknown prior anti‐VEGF treatment history due to participation in a double‐masked randomized study were excluded from the variable ‘number of different agents used before the switch’ and from association analyses involving previous treatment. However, since other anatomical and outcome parameters were available, these eyes were retained in the overall analysis for variables where complete data existed.

All statistical tests were two‐tailed, and a *p*‐value < 0.05 was considered statistically significant. Analyses were conducted using sas software version 9.4 (SAS Institute Inc., Cary, NC, USA).

## 3 RESULTS

### 3.1 Study population and baseline characteristics

Out of 1049 patients screened for inclusion, a total of 167 patients (181 eyes) with nAMD who were previously treated with anti‐VEGF therapy and switched to aflibercept 8 mg met the eligibility criteria and were included in the final analysis. The mean age of the switch population was 80.4 ± 8.5 years (range 55–102 years), and 67.7% were female. The median follow‐up duration was 12.9 weeks (range 4.1–48.1 weeks). On average, 2.1 ± 1.5 injections of aflibercept 8 mg were administered per eye during the study period (range 1–9 injections), corresponding to a total of 415 injections across the cohort.

Prior to the switch, patients had received a median of 9 anti‐VEGF injections (IQR 2–57), with the most common preceding therapy being aflibercept 2 mg (72.5%), followed by bevacizumab (12.4%), faricimab (12.4%), brolucizumab (2.2%) and dexamethasone implant (0.6%). The average number of prior different anti‐VEGF medications was 1.43 ± 0.62, with a median of 1.0 (range 1–3) (Table 1).

**TABLE 1:** Baseline and post‐treatment characteristics of eyes treated with aflibercept 8 mg.

Variable	Previously treated nAMD eyes (*N* = 181)
BCVA (baseline)	0.46 ± 0.31 0.40 (0.00–1.30) *n* = 122
BCVA (post‐treatment)	0.47 ± 0.37 0.30 (0.00–1.52) *n* = 76
Change in BCVA	−0.03 ± 0.17 0.00 (−0.51 to 0.52) *n* = 53
CRT (baseline)	249.4 ± 67.7 237 (126–524) *n* = 181
CRT (post‐treatment)	229.9 ± 52.9 226 (121–514) *n* = 181
Change in CRT	−19.5 ± 47.2 –6 (−293 to 113) *n* = 181
PED (baseline)	
No	94 (51.9%)
Yes	87 (48.1%)
Height of PED (μm) (baseline)	249.3 ± 140.2 198 (56–714) *n* = 87
Height of PED (μm) (post‐treatment)	211.9 ± 127.6 185 (0–632) *n* = 87
Change in height of PED (μm)	−37.4 ± 68.4 –13 (−334 to 46) *n* = 87
Residual fluid (baseline)	
No	55 (30.4%)
Yes	126 (69.6%)
Residual fluid (post‐treatment)	
No	101 (56.4%)
Yes	78 (43.6%)
Intraretinal fluid (baseline)	
No	119 (65.7%)
Yes	62 (34.3%)
Intraretinal fluid (post‐treatment)	
No	146 (80.7%)
Yes	35 (19.3%)
Subretinal fluid (baseline)	
No	85 (47.0%)
Yes	96 (53.0%)
Subretinal fluid (post‐treatment)	
No	120 (66.3%)
Yes	61 (33.7%)
Both types of fluid (baseline)	
No	151 (83.4%)
Yes	30 (16.6%)
Both types of fluid (post‐treatment)	
No	165 (91.2%)
Yes	16 (8.8%)
Maximal length of extension treatment interval (weeks) with dry macula	9.43 ± 3.96 9 (4–21) *n* = 114
Maximal length of extension treatment interval (weeks) with dry macula/follow‐up time	0.67 ± 0.32 0.67 (0.09–1.00) *n* = 114
Time to reappearance of fluid	11.40 ± 4.24 10 (6–19) *n* = 15
Time to reappearance of fluid/follow‐up time	0.48 ± 0.55 0.28 (0.14–2.33) *n* = 15
Number of previous anti‐VEGF medications	1.4 ± 0.6 1.0 (1.0–3.0) *n* = 168

*Note*: Continuous variables were described by mean ± SD, median (range), number of observations and categorical variables by frequency and percentage.

Abbreviations: μm, micrometres; BCVA, best‐corrected visual acuity; CRT, central retinal thickness; nAMD, neovascular age‐related macular degeneration; PED, pigment epithelial detachment; VEGF, vascular endothelial growth factor.

### 3.2 Visual outcomes

**FIGURE 1:**
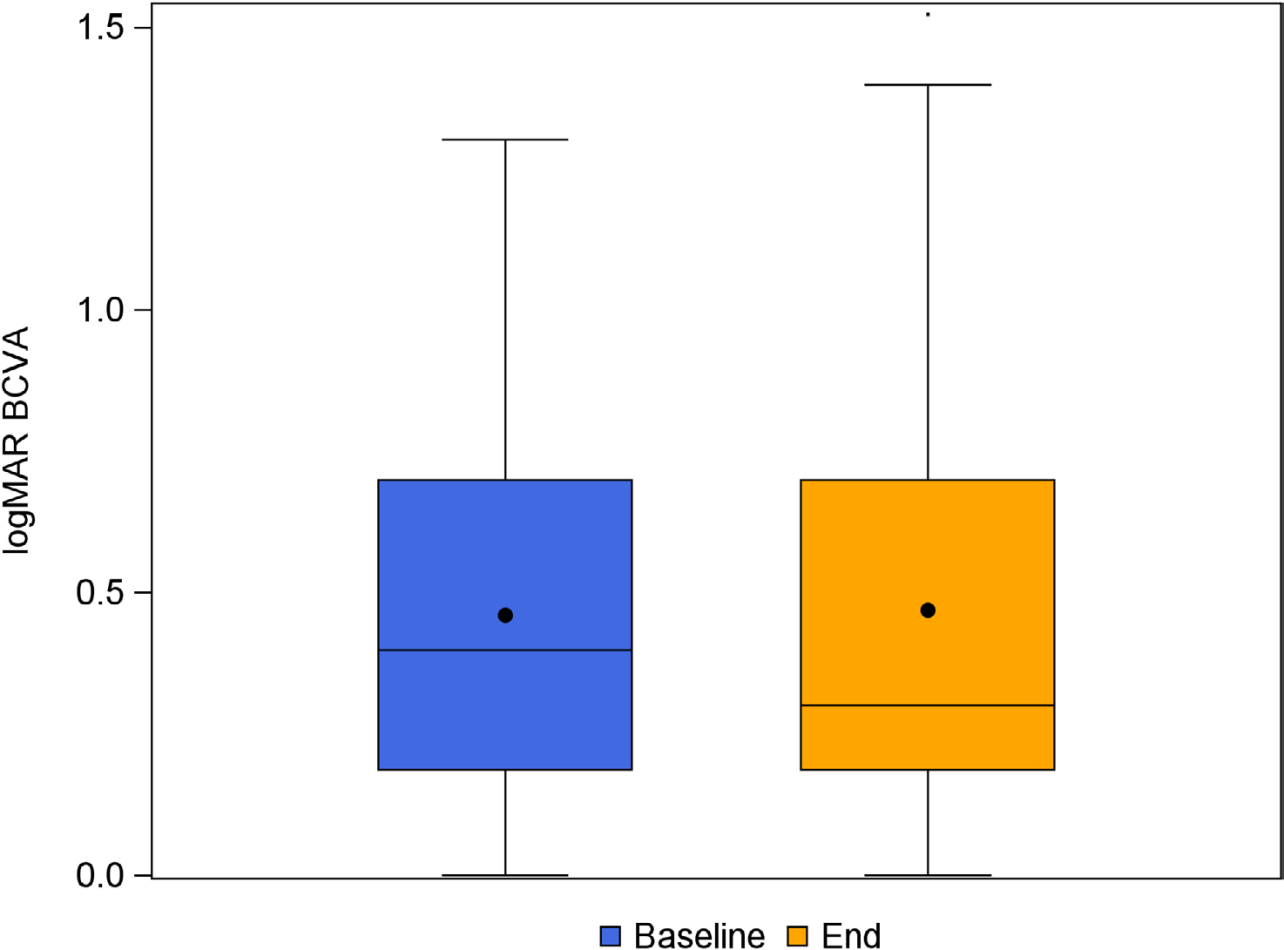
Change in best‐corrected visual acuity (BCVA) expressed as logMAR after switching to aflibercept 8 mg. Boxplot displays median, interquartile range and outliers for the change in logMAR BCVA from baseline to follow‐up. No statistically significant change was observed (*p* = 0.18).

BCVA remained stable following the switch to aflibercept 8 mg. Mean baseline BCVA was 0.46 ± 0.31 logMAR, and post‐treatment BCVA was 0.47 ± 0.37 logMAR, with no statistically significant difference (*p* = 0.18). Among evaluable eyes (*n* = 53), the mean change in BCVA was −0.03 ± 0.17 logMAR (median 0.00; range −0.51 to +0.52) (Figure 1). Multivariable regression analysis showed no significant associations between the change in BCVA and baseline factors, including age, sex, prior treatment agent or number of aflibercept 8 mg injections. To evaluate the representativeness of eyes with available BCVA data, dropout analyses comparing included and excluded eyes were performed. These analyses revealed some differences in baseline CRT, presence and height of PED, and post‐treatment residual and subretinal fluid (see Tables S1 and S2). Importantly, this limitation applied only to BCVA data; OCT‐based anatomical outcomes, including fluid status, were complete for nearly all eyes.

### 3.3 Anatomical outcomes

**FIGURE 2:**
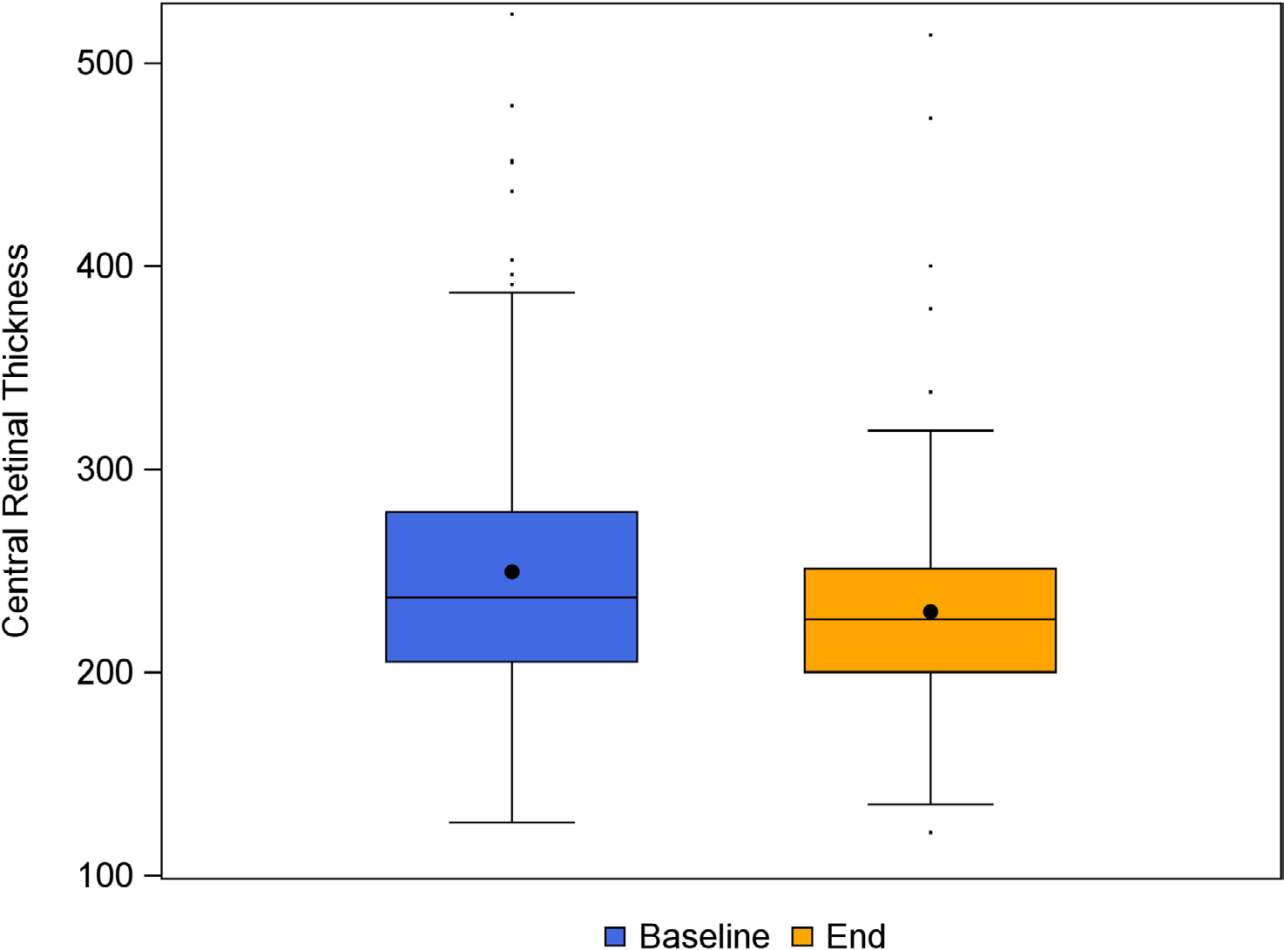
Change in central retinal thickness (CRT) following the switch to aflibercept 8 mg. The boxplot displays the median, interquartile range (IQR) and individual outliers for the change in CRT from baseline to follow‐up. A statistically significant anatomical improvement was observed (*p* < 0.001).

Significant anatomical improvements were observed following the switch. The mean CRT decreased from 249.4 ± 67.7 μm at baseline to 229.9 ± 52.9 μm at follow‐up (*p* < 0.001), with a mean change of −19.5 ± 47.2 μm (Figure 2). In the mixed‐effects model, baseline CRT was a strong predictor of CRT reduction, with a mean decrease of 44.1 μm per 100 μm increase in baseline CRT (95% CI: −53.0 to −35.2; *p* < 0.001).

Among eyes with fibrovascular PED within the central 1500 μm (*n* = 87), the mean PED height decreased from 249.3 ± 140.2 μm to 211.9 ± 127.6 μm (*p* < 0.001). The average reduction in PED height was −37.4 ± 68.4 μm (median − 13; range −334 to +46). Greater baseline PED height was associated with larger reductions post‐treatment (*β* = −0.22 per μm (95% CI: −0.31 to −0.13); *p* = 0.002).

### 3.4 Fluid resolution

The proportion of eyes with IRF decreased from 34.3% at baseline to 19.3% at follow‐up (*p* < 0.001). SRF was present in 53.0% of eyes at baseline and decreased to 33.7% after treatment (*p* < 0.001). Furthermore, the proportion of eyes with both IRF and SRF declined significantly from 16.6% to 8.8% (*p* = 0.0026). Fluid resolution was not significantly associated with age, sex, treatment duration or number of aflibercept 8 mg injections.

### 3.5 Treatment interval dynamics

The median maximal dry interval achieved after switching to aflibercept 8 mg was 9 weeks (range 4–21 weeks), with a mean of 9.43 ± 3.96 weeks. Among eyes that experienced recurrence of fluid, the median time to fluid reappearance was 10 weeks (range 6–19), with a mean of 11.40 ± 4.24 weeks.

Poisson regression identified higher baseline CRT as a significant negative predictor of interval extension: Each 100 μm increase in CRT was associated with a 33% reduction in the likelihood of reaching longer dry intervals (RR = 0.67; 95% CI: 0.57–0.79; *p* < 0.001). The number of aflibercept 8 mg injections was also inversely associated with interval extension (RR = 0.61; *p* < 0.001).

In the subgroup of eyes with ≥12 weeks of follow‐up (*n* = 121), results for maximal dry interval and fluid recurrence were consistent with those in the overall cohort, confirming the robustness of our conclusions despite variability in follow‐up times.

### 3.6 Interval extension analysis

In the full analysis set (*n* = 85), the median maximal interval length increased by 2 weeks after switching to aflibercept 8 mg, with a mean change of 1.27 ± 4.24 weeks (range −13 to +9 weeks; *p* = 0.0009). Improvement in interval length was observed in 55 eyes (64.7%), no change in 5 eyes (5.9%) and worsening in 25 eyes (29.4%). Among eyes with no fluid at baseline, interval extension was more pronounced, with a mean change of 3.68 ± 2.08 weeks (*p* < 0.0001), while eyes with baseline fluid showed no statistically significant interval change (mean − 0.87 ± 4.54 weeks; *p* = 0.30). In the subgroup with at least 16 weeks of follow‐up (*n* = 30), a similar trend was observed: Mean interval extension was 1.30 ± 3.72 weeks overall (*p* = 0.08), with statistically significant interval prolongation in eyes without fluid before the switch (mean 3.15 ± 2.58 weeks; *p* = 0.0015) and no statistically significant change in those with baseline fluid (mean −0.12 ± 3.89 weeks; *p* = 0.77).

### 3.7 Association analyses with previous treatment

Analyses of the switch population revealed no statistically significant associations between BCVA change and prior anti‐VEGF therapy (aflibercept 2 mg, bevacizumab, faricimab or brolucizumab; *p* = 0.58). Similarly, changes in CRT and PED height did not differ significantly across treatment groups (*p* = 0.24 for CRT and *p* = 0.32 for PED height). Furthermore, the type of previous treatment was not associated with fluid resolution at follow‐up, including IRF, SRF or overall fluid status. Finally, the maximal dry macula interval achieved was not significantly influenced by the previous anti‐VEGF agent.

### 3.8 Safety

Aflibercept 8 mg was well tolerated in this cohort. Only one eye (0.6% of eyes; 0.2% per injection) experienced mild anterior uveitis (iritis), which was resolved with topical corticosteroids. There were no reported cases of vitritis, vasculitis, panuveitis, IOP elevation (documented or symptom‐related) or endophthalmitis during the follow‐up period.

## 4 DISCUSSION

Our study reflects the realities of treating neovascular AMD in a high‐volume, routine clinical setting, where treatment strategies and protocols can evolve rapidly based on new evidence, logistical factors and economic considerations. During the observation period, our centre employed a treat‐and‐extend regimen, typically initiating therapy with two to three injections at intervals determined by disease activity, fluid status on OCT and previous response to other anti‐VEGF treatments. Interval adjustments were individualized; for example, if patients were dry but required short intervals to remain stable (e.g. every 4 weeks), gradual extension by approximately 2 weeks was attempted; if eyes were not dry, the same interval was maintained. Decisions regarding interval adjustments were guided primarily by OCT‐based anatomical outcomes, consistent with routine practice where structural imaging takes precedence over frequent VA testing to efficiently manage patient flow. Importantly, a protocol change occurred during the study period, in which all patients previously receiving aflibercept 2 mg were switched to aflibercept 8 mg, regardless of their disease status at the time, as part of a centre‐wide decision to streamline treatment logistics and align with updated purchasing agreements. These factors contributed to the heterogeneity in baseline fluid status and follow‐up durations observed in our cohort, but also underscore the relevance of our findings to real‐world clinical practice.

In patients with nAMD who were switched to aflibercept 8 mg, we observed significant anatomical improvements and stable VA outcomes over a median follow‐up of approximately 13 weeks. Although functional gains were limited, visual stability itself represents a positive outcome in a previously treated population with chronic disease, where progressive deterioration is expected without effective intervention.

This observation aligns with findings from Bala et al., who reported stable VA in a similar cohort of previously treated nAMD patients switched to aflibercept 8 mg. Specifically, they noted that mean vision remained approximately 62 ETDRS letters at both baseline and final follow‐up, indicating no significant change (*p* = 0.93) (Bala et al., 2025).

The reduction in CRT, IRF, SRF and PED height following the switch is consistent with earlier reports suggesting that aflibercept 8 mg enhances anatomical control in patients with a suboptimal response to standard‐dose anti‐VEGF therapy. Bala et al. (2025) observed a decrease in IRF prevalence from 53.9% to 39.3% and SRF from 54.3% to 41.1% over approximately six months, which mirrors our findings of a 15% and 20% absolute reduction in IRF and SRF prevalence, respectively.

Despite these anatomical improvements, BCVA remained unchanged among most patients. This anatomical–functional dissociation has been described in previous studies and may reflect irreversible damage from chronic exudation, outer retinal atrophy or fibrosis. Guymer et al. (2019) similarly reported that tolerating SRF in nAMD treated with ranibizumab, using a treat‐and‐extend regimen, did not adversely affect VA outcomes, suggesting that anatomical improvements do not always correlate directly with functional gains.

We documented the maximal treatment interval with a dry macula and the time to fluid recurrence. The median maximal dry interval achieved was nine weeks, and the median time to fluid reappearance was ten weeks among those with recurrence. These results reflect expected outcomes in a challenging switch population and highlight the importance of tailoring follow‐up based on disease activity. Interestingly, we found that higher baseline CRT was associated with shorter achievable dry intervals, reinforcing the need for closer monitoring in eyes with more active disease at the time of switching. In the subgroup of eyes with ≥12 weeks of follow‐up, results for maximal dry interval and fluid recurrence were consistent with those in the overall cohort, confirming the robustness of our conclusions despite variability in follow‐up times.

Our analysis comparing treatment intervals before and after switching demonstrated a statistically significant interval extension overall, particularly in eyes that were dry at baseline. This finding supports the potential of aflibercept 8 mg to enable longer treatment intervals in stable, previously treated eyes, which may help reduce treatment burden.

Association analyses showed that outcomes were not significantly related to the type of prior anti‐VEGF agent. Patients who switched from aflibercept 2 mg, bevacizumab, faricimab or brolucizumab demonstrated comparable changes in BCVA, CRT, PED height and fluid resolution. This suggests that aflibercept 8 mg can be beneficial regardless of previous therapy, supporting its use as a flexible switch option (Bala et al., 2025; Sambhara et al., 2025).

One patient with a confirmed diagnosis of exudative neovascular AMD received a dexamethasone 0.7 mg intravitreal implant off‐label at the time of switch, due to persistent macular fluid despite multiple prior anti‐VEGF agents. In this challenging case, the treating physician suspected an inflammatory component contributing to refractory disease activity and initiated corticosteroid therapy as a last resort to achieve anatomical improvement. While this reflects real‐world management decisions in challenging cases, it may introduce some heterogeneity to the cohort.

In terms of safety, aflibercept 8 mg was well tolerated, with only one mild case of anterior uveitis reported, and no instances of vitritis, vasculitis, panuveitis or endophthalmitis. This aligns with the favourable safety profile observed in previous trials and post‐marketing surveillance. The phase 3 PULSAR trial reported low rates of intraocular inflammation with aflibercept 8 mg, with no cases of retinal vasculitis or occlusive retinitis observed in the aflibercept 8 mg group (Lanzetta et al., 2024). While rare cases of inflammation‐associated vasculitis have been reported post‐approval, such complications appear infrequent and were not observed in our cohort. For instance, Matsumoto et al. (2024) reported three cases (8.6%) of non‐infectious intraocular inflammation associated with retinal vasculitis in a cohort of 35 eyes treated with aflibercept 8 mg, all of which resolved without vision loss. By contrast, Hashiya et al. (2025) described a case of severe inflammation, including retinal vasculitis and vascular occlusion, in a patient with a prior history of intraocular inflammation, resulting in poor visual outcome despite steroid treatment (Hashiya et al., 2025; Matsumoto et al., 2024).

One notable strength of this study is that it represents one of the early publications providing real‐world evidence for the use of aflibercept 8 mg in previously treated nAMD patients. To the best of our knowledge, this is the first real‐world study of aflibercept 8 mg conducted in the Nordic countries. This adds valuable insight into the performance of this treatment option in broader, diverse and less protocol‐driven clinical settings typical of real‐world practice in Scandinavia.

While our findings offer important insights into the practical use of aflibercept 8 mg in previously treated nAMD patients, several limitations should be considered. Due to successive enrolment and variability typical of routine clinical practice, follow‐up durations varied substantially across patients, which may introduce heterogeneity in interval‐related outcomes. We addressed this limitation by adjusting for individual follow‐up times in key analyses and performing subgroup analyses for eyes with longer follow‐up, which confirmed the consistency of our findings.

As IOP measurements were performed only in symptomatic cases, reflecting standard clinical workflow in our clinic, asymptomatic IOP elevations may have gone undetected, potentially leading to underestimation of the true incidence of IOP‐related adverse events.

During the study period, our centre transitioned from using aflibercept 2 mg to aflibercept 8 mg for all patients, both new and ongoing treatments. As a result, many baseline OCT scans were performed while patients were still receiving aflibercept 2 mg, which did not provide a valid pre‐switch imaging assessment. This practical change, driven by logistical and treatment strategy considerations, led to the exclusion of a large number of patients. Indeed, this was one of the most common causes of exclusion during our screening process, with only 167 of 1049 screened patients ultimately included in the study. This experience highlights the complexities of conducting real‐world retrospective studies during periods of evolving treatment protocols.

Due to routine clinical protocols prioritizing OCT‐based structural assessment over functional testing, VA measurements were not consistently recorded at every evaluation visit. This approach, reflecting routine clinical practice in a high‐volume clinic, resulted in missing BCVA data for many eyes. However, anatomical outcomes, especially fluid status assessed by OCT, were available for nearly the entire cohort. Although dropout analyses indicated some anatomical differences between included and excluded eyes, the limited availability of VA data remains a limitation of this study.

The relatively short observation periods in some eyes, due to staggered enrolment and real‐world variability, may have limited our ability to fully characterize treatment interval extension. This underscores the challenges of evaluating interval durability in retrospective, real‐world switch studies. Furthermore, as this study reports early real‐world outcomes following the introduction of aflibercept 8 mg at our centre, longer‐term follow‐up data were not yet available during the observational period. This inherent limitation should be considered when interpreting our findings in the context of randomized controlled trials, which typically include standardized treatment intervals and observation periods in treatment‐naïve patients.

In conclusion, switching to aflibercept 8 mg in previously treated nAMD patients was associated with significant anatomical benefits, stable visual outcomes and a favourable short‐term safety profile. While functional gains were limited, our analysis comparing pre‐ and post‐switch intervals demonstrated that many eyes achieved extended treatment intervals, suggesting the potential to reduce treatment burden. Nonetheless, variability in follow‐up durations and the observational nature of the study highlight the need for longer follow‐up to confirm the durability of these effects and to capture potential late‐onset safety outcomes. These findings suggest that aflibercept 8 mg may be an effective strategy for optimizing disease control in routine management of previously treated nAMD patients.

## CONFLICT OF INTEREST STATEMENT

Imadeddin Abu Ishkheidem and Martin Breimer have served as consultants to Bayer, including giving a webinar about aflibercept 8 mg. The remaining authors declare no financial or proprietary interests in any material or method mentioned.

## Supporting information


Table S1.
