# Supplementary material for: Real‐world outcomes of aflibercept 8 mg in patients previously treated for neovascular age‐related macular degeneration
Source: Acta Ophthalmol. 2025 Sep 8;104(3):296–304. doi: 10.1111/aos.17590 (PMC13058681; doi:10.1111/aos.17590)
Supplement: Supplementary file 1 — Table S1. [file AOS-104-296-s001.docx]

# **Supplementary Table S1.** Patient characteristics overall and by missingness in change in best corrected visual acuity (BCVA)

| **Variable** | **Not missing change in BCVA (N=49)** | **Missing change In BCVA (N=118)** | **P-value** | **Overall (N=167)** |
| --- | --- | --- | --- | --- |
| **Age** | 79.8±8.9 80.0 (55.0 - 102.0) n=49 | 80.6±8.3 80.0 (58.0 - 99.0) n=118 | 0.59 | 80.4±8.5 80.0 (55.0 - 102.0) n=167 |
| **Treatment duration (weeks)** | 15.1±7.8 12.9 (5.0 - 46.7) n=49 | 17.2±10.7 12.9 (4.1 - 48.1) n=118 | 0.80 | 16.5±9.9 12.9 (4.1 - 48.1) n=167 |
| **Sex** |  |  | 0.08 |  |
| **Male** | 11 (22.4%) | 43 (36.4%) |  | 54 (32.3%) |
| **Female** | 38 (77.6%) | 75 (63.6%) |  | 113 (67.7%) |

# **Supplementary Table S2.** Eye characteristics overall and by missingness in change in best corrected visual acuity (BCVA)

| **Variable** | Not missing change in BCVA (N=49) | Missing change in BCVA (N=118) | | p-value | Overall (N=167) |
| --- | --- | --- | --- | --- | --- |
| **BCVA (Baseline)** | 0.50±0.35 0.40 (0.00–1.30) n=53 | 0.43±0.27 0.40 (0.00–1.00) n=69 | 0.19 | | 0.46±0.31 0.40 (0.00–1.30) n=122 |
| **BCVA (Posttreatment)** | 0.47±0.37 0.30 (0.00–1.52) n=53 | 0.47±0.35 0.30 (0.10–1.22) n=23 | 0.96 | | 0.47±0.37 0.30 (0.00–1.52) n=76 |
| **Change in BCVA** | -0.03±0.17 0.00 (-0.51–0.52) n=53 | — | — | | -0.03±0.17 0.00 (-0.51–0.52) n=53 |
| **CRT (Baseline) (µm)** | 268.0±79.3 244 (166–524) n=53 | 241.7±61.0 236 (126–452) n=128 | 0.033 | | 249.4±67.7 237 (126–524) n=181 |

Data presented as mean ± SD, median (range), number (percentage). Abbreviations: BCVA, best corrected visual acuity; CRT, central retinal thickness; PED, pigment epithelial detachment; µm, micrometers.
